# Enterovirus-Infected β-Cells Induce Distinct Response Patterns in BDCA1^+^ and BDCA3^+^ Human Dendritic Cells

**DOI:** 10.1371/journal.pone.0121670

**Published:** 2015-03-25

**Authors:** Barbara M. Schulte, Paul R. Gielen, Esther D. Kers-Rebel, Gerty Schreibelt, Frank J. M. van Kuppeveld, Gosse J. Adema

**Affiliations:** 1 Department of Tumor Immunology, Radboud university medical center, Radboud Institute for Molecular Life Sciences, Nijmegen, The Netherlands; 2 Virology Division, Department of Infectious Diseases and Immunology, Faculty of Veterinary Medicine, Utrecht University, Utrecht, The Netherlands; University of Bergen, NORWAY

## Abstract

Enteroviruses often cause mild disease, yet are also linked to development of autoimmune diabetes. Dendritic cells (DCs) shape both innate and adaptive immune responses, including anti-viral responses. How different human DC subsets shape anti-viral responses, whether they have complementary or overlapping functions and how this relates to autoimmune responses is largely unknown. We used enterovirus-infected β-cells and freshly isolated human myeloid DC (mDC) subsets as a model for autoimmune type 1 diabetes. Our data show that both the BDCA1^+^ and BDCA3^+^ mDC subsets engulf mock- as well as virus-infected β-cells, albeit BDCA1^+^ mDCs are more efficient. Uptake of enterovirus-infected, but not mock-infected cells, activated both DC subsets as indicated by the induction of co-stimulatory molecules and secretion of type I and type III interferons. Both subsets produced similar amounts of interferon-α, yet the BDCA3^+^ DC were superior in IFN-λ production. The BDCA1^+^ mDCs more strongly upregulated PD-L1, and were superior in IL-12 and IL-10 production as compared to the BDCA3^+^ DC. Despite lack of IL-12 production by the BDCA3+ DC, both BDCA1^+^ and BDCA3^+^ DCs activated T cells in allogeneic mixed lymphocyte reaction towards a Th1-type reactivity while suppressing Th2-associated cytokines.

## Introduction

The immunopathology for many autoimmune disorders remains unclear, but infections in general and viral infections in particular have been hypothesized to accelerate or even trigger autoimmune diseases [1–5]. Viruses can directly infect and kill target cells, or alternatively, can induce inflammation and autoimmunity through indirect immune mechanisms e.g. via bystander immune activation [6, 7]. Antigen-presenting dendritic cells (DCs) are key modulators of immune homeostasis and induce immune responses against pathogens while maintaining tolerance to self to prevent autoimmunity [8, 9]. Here, we study early DC immune activation upon encounter with virus-infected β-cells using fresh human myeloid DCs (mDCs) instead of the monocyte-derived DCs generated *in vitro*. This setting better mimics the physiological situation during enterovirus infections and may resemble the situation during type 1 diabetes pathogenesis. We used coxsackie B3 virus (CVB3), an enterovirus belonging to the human enterovirus B (HEV-B) viruses which are associated with type 1 diabetes development [5, 6, 10]. HEV-B viruses are small RNA viruses that usually cause mild or asymptomatic infections in the gastrointestinal tract, yet severe infections can occur resulting in e.g. myocarditis or encephalitis. Furthermore, HEV-Bs have been associated with chronic autoimmune diseases such as type 1 diabetes and myocarditis [5, 11] and, importantly, they have a tropism for islets of Langerhans [12–14].

Dendritic cells (DCs) shape both the innate and adaptive immune responses, and as such are implicated in the pathogenesis of most autoimmune diseases [8]. Furthermore antigen-presenting cells (APCs) such as DCs are critical during the induction of virus-induced autoimmunity [15]. Moreover, differences in DC-number and function have been reported between type 1 diabetes-patients and healthy donors [16–20], although some studies contradict each other indicating that further research is required. In order to respond adequately DCs sample their microenvironment and phagocytose foreign material as well as apoptotic and necrotic cells [21]. DCs express specialized receptors known as pattern recognition receptors (PRRs) which bind pathogen-associated molecular patterns (PAMPs). Upon triggering of these receptors the DCs become activated and initiate immune responses [22]. DCs modulate innate immunity, e.g. via the production of type I interferons (IFNs), as well as adaptive immune responses, e.g. by antigen presentation, costimulation and production of cytokines and chemokines which results in activation and skewing of (naïve) T and B cells. Most knowledge on human DC function originates from studies using monocyte-derived DCs; however, these *in vitro* differentiated DCs differ markedly from naturally occurring DCs [22–24].

In recent years 2 subsets of naturally occurring human blood derived myeloid DCs (mDCs) have been described, BDCA1^+^/CD1c^+^ mDCs and BDCA3^+^/CD141^+^ mDCs [25]. These subsets differ in their expression of cell surface markers, Toll-like receptors (TLRs) and other PRRs, such as RIG-I-like receptors [25–28]. The BDCA3^+^ mDCs are reported to be more efficient in cross-presenting antigen to CD8^+^ cytotoxic T lymphocytes (CTLs) compared to BDCA1^+^ mDCs [27, 29–31]. Only few functional studies on responses of human BDCA1^+^ and BDCA3^+^ DCs have been published due to the low frequency of these cells in human blood (BDCA1^+^ and BDCA3^+^ DCs resemble approximately 0.3–1% and 0.04% of human peripheral blood mononuclear cells (PBMCs)). Studies so far mainly focused on the response upon stimulation with a high amount of isolated TLR-ligands [28–30], a situation that does not well resemble physiological conditions where infected and damaged cells are encountered by DCs. We studied the response of BDCA1^+^ and BDCA3^+^ mDC subsets from healthy donors upon encounter of enterovirus-infected cells to determine overlapping and complementary functions and discuss possible implications for induction of autoimmune reactions.

Our data show that although BDCA1^+^ and BDCA3^+^ mDC subsets have distinct response patterns upon exposure to enterovirus-infected β-cells, they both promote Th1 responses that could favor the induction or maintenance of β-cell autoimmune reactivity.

## Materials and Methods

### Ethics Statement

Buffycoats or leukapheresis products were obtained from healthy volunteers after written consent according to institutional guidelines and the Declaration of Helsinki and were obtained via Sanquin Blood Bank, Nijmegen, The Netherlands. Blood products were released anonymized to laboratory personnel.

### Isolation and culture of cells

BDCA1^+^ mDCs were isolated by magnetic-activated cell sorting (MACS) using a BDCA-1 isolation kit, according to the manufacturer’s instructions (Miltenyi Biotec). In short, peripheral blood mononuclear cells (PBMCs) were isolated from buffy coat/leukapheresis products using a Ficoll gradient, washed, followed by CD19^+^ cell-depletion to deplete B cells and subsequently positively selected for BDCA1^+^ cells. To obtain peripheral blood leukocytes (PBLs), monocytes were depleted from PBMCs by adherence to plastic culture flasks. BDCA3^+^ mDCs were subsequently isolated from PBLs by positive selection using a BDCA3^+^ mDC isolation kit as described [27]. Cells were routinely up to 95% pure, as assessed by double staining BDCA1-APC/CD11c-PE (Miltenyi Biotec, and BD Pharmingen, respectively) or BDCA3-APC/CD11c-PE. mDCs were cultured in X-Vivo 15 (Lonza) supplemented with 5ng/ml GM-CSF (Strahtman) and were used for experiments immediately after isolation. DCs were cultured 0.3x10^6^ cells in a 24-well culture plate in 400μl medium, or scaled up or down according to number of cells available.

Min6 insulinoma cells [32] were a gift from dr. Per Bendix Jeppesen and were cultured in Dulbecco’s modified Eagle’s medium (DMEM)(Gibco) supplemented with 15% FCS, 0.5% antibiotic/antimycotic and 50 μmol/l β-mercaptoethanol (complete DMEM) at 37°C in 5% CO2. Medium was refreshed every other day.

### Virus propagation, purification and infection of β-cells

CVB3 Nancy (CVB3) was kindly provided by R. Kandolf (University of Tübingen, Germany). Production of virus stocks and virus titrations were performed as described [33]. When used for flowcytometry purposes, cells were labeled with PKH67 (Sigma Aldrich) prior to infection. Min6 cells were infected at a multiplicity of infection (MOI) of 15 in complete DMEM [34]. After 1 hour incubation at 37°C cells were washed to remove excess virus and were cultured in complete DMEM for 48 hours before harvesting the cells for co-culture with human BDCA1^+^ or BDCA3^+^ mDCs.

### mDC stimulation

mDCs were left unstimulated, or stimulated with poly I:C (Enzo Life Sciences)(20μg/ml), or were co-cultured in a 1:1 ratio with mock- or CVB3-infected Min6 cells. Alternatively, mDCs were exposed to CVB3 (MOI of 50) in X-Vivo15. Supernatant and cells were harvested at indicated times for mRNA isolation, flow cytometry or ELISA/bead array, or used in allogeneic mixed lymphocyte reaction (MLR).

### RNA isolation and quantitative PCR (qPCR)

RNA isolations were done using the ZR RNA isolation kit (Zymo Research) and reverse-transcribed into cDNA as described [35]. Alternatively, total RNA was isolated with RNeasy MiniPrep Kit (Qiagen Benelux). The optional on-column DNAse treatment was applied. RNA was transcribed into cDNA with the RT2 First Strand cDNA Kit (SABiosciences, Qiagen Benelux) according to manufacturer’s instructions. mRNA levels for the genes of interest were determined by quantitative PCR (qPCR) with a Biorad CFX apparatus (Biorad) using SYBR Green (Applied Biosystems). Analysis was done using Biorad CFX-1.6 software. Primer sequences are available on request and most are from the Primer Bank database [36].

### Flow cytometry

Cells were harvested with cold PBS at indicated time points, washed twice in PBS and stained with fixable viability dye (eBioscience) for 30 minutes on ice. Subsequently, cells were stained as described [33] with conjugated antibodies directed against cell surface markers or corresponding isotypes. CD80-PeCy7, CD86-PeCy7, PD-L1-PE and PD-L2-PE were all from BD Pharmingen; ICOS-L-PerCP was from R&D systems and B7-H3-Alexa488 and B7-H4-Alexa488 were from AbD Serotec. In Min6 co-cultures, expression of co-stimulatory molecules was analyzed on CD11c-APC^high^ (BD) or BDCA3-APC (Miltenyi) expressing mDCs for BDCA1 and BDCA3 mDCs, respectively. Cells were analyzed on a CyAn Flow cytometer (Beckman Coulter) and data was analyzed using FlowJo software, gated on living cells. Pulse width analysis was included to exclude doublets and only analyze single cells. An example of the gating strategies used is shown in S1 Fig.

### Allogeneic MLR

DCs were harvested using cold PBS after overnight (o/n) stimulation and co-cultured with allogeneic peripheral blood lymphocytes in a 1:10 DC:PBL ratio using 1x10^5^ PBLs per well in a 96-well U-bottom plate. After 4 days of culture T cell proliferation was assessed by adding 1 μCi/well of tritiated thymidine for 16 hours. Incorporated thymidine was measured in a β-counter.

### ELISA and bead array

IFN-λ1, IL-10 and IL-27 production was analyzed with a human IFN-lambda 1 ELISA (IL-29) kit, human IL-10 ELISA kit and human IL-27 ELISA kit (all from eBioscience) respectively. Millipore human 22-plex was used to determine multiple cytokine and chemokine levels produced (the following cytokines were measured: Flt3-L, Fractalkine, G-CSF, IFNα2, IFNγ, IL-1α, IL-1β, IL-1ra, IL-6, IL-12p70, IL-13, IL-15, IL-17, IP-10, MCP1, MCP3, MDC, MIP1α, MIP1β, RANTES, TGFα, TNFα). Data analysis was performed with Bio-Plex Manager software (Bio-Rad Laboratories). T helper cell cytokines were determined by 13-plex Th1/Th2/Th9/Th17 bead array (BenderMed systems).

### Statistical analysis

Statistical analysis was performed using ANOVA followed by post-hoc Tukey test as indicated. A p-value <0.05 was considered significantly different.

## Results

### Human BDCA1^+^ and BDCA3^+^ mDCs phagocytose mock- and CVB-infected murine insulinoma cells

**Fig 1:**
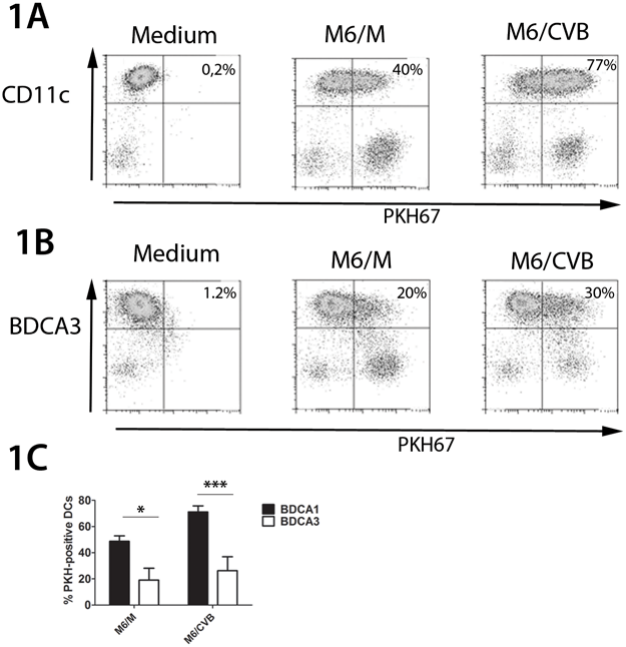
BDCA1^+^ mDCs more efficiently phagocytose murine Min6 cells compared to BDCA3^+^ mDCs. **A**) BDCA1^+^ mDCs or **B**) BDCA3^+^ mDCs were co-cultured overnight (o/n) with PKH67-labeled mock- or CVB-infected Min6 cells (M6/M and M6/CVB, respectively) stained for the CD11c or BDCA3 and analyzed by flow cytometry on viable, single cells (See S1 Fig). Percentages in upper right corner represent the percentage of DCs that has engulfed Min6 material. This was calculated as follows: percentage PKH^+^ DCs/total DCs (i.e. total CD11c positive cells or total BDCA3 positive cells) **C**) DCs were analyzed as in A) and B), shown is average + SEM for >3 donors. * p<0.05, *** p<0.001 determined by ANOVA and post-hoc Tukey test.

To investigate whether freshly isolated mDC subsets phagocytose pancreatic β-cells, they were co-cultured with PKH67-labeled Min6-cells that were mock- or CVB3-infected [34]. During physiological circumstances DCs will encounter infected cells at different stages of infection. Therefore, virus-infected cells were infected 48 hours prior to co-culture with DCs. In this setting a heterogeneous population of Min6 insulinoma cells is present showing cytopathic effects in approximately 80% of the cells (S2 Fig). Mock- and CVB-infected cells were efficiently taken up by BDCA1^+^ mDCs, resulting in an average of 50 to 70% of PKH^+^ BDCA1^+^ DCs, respectively (Figs. 1A and C). Uptake of CVB-infected cells was somewhat more efficient than uptake of mock-infected cells, although this did not reach statistical significance (Fig. 1C). BDCA3^+^ mDCs also engulfed mock-and CVB-infected Min6 material (Figs. 1B and C), although a lower proportion of BDCA3^+^ DCs engulfed Min6 material. For example, approximately 70% of BDCA1^+^ DCs had engulfed CVB-infected Min6 material after overnight (o/n) co-culture, compared to approximately 30% of BDCA3^+^ mDCs (Fig. 1C). BDCA3^+^ DCs that did engulf labeled Min6 material also had a lower mean fluorescent intensity than BDCA1^+^ mDCs further suggesting uptake by BDCA1^+^ mDCs is more efficient (Fig. 1B). Confocal analysis confirmed the uptake of β-cell material by showing the presence of PKH-labeled Min6 material within DCs (S3 Fig). Thus, both blood mDC subsets can engulf mock- and CVB-infected β-cells, although BDCA1^+^ mDCs more efficiently than BDCA3^+^ mDCs.

### BDCA1^+^ and BDCA3^+^ mDC produce IFNs after uptake of CVB3-infected, but not mock-infected β-cells

**Fig 2:**
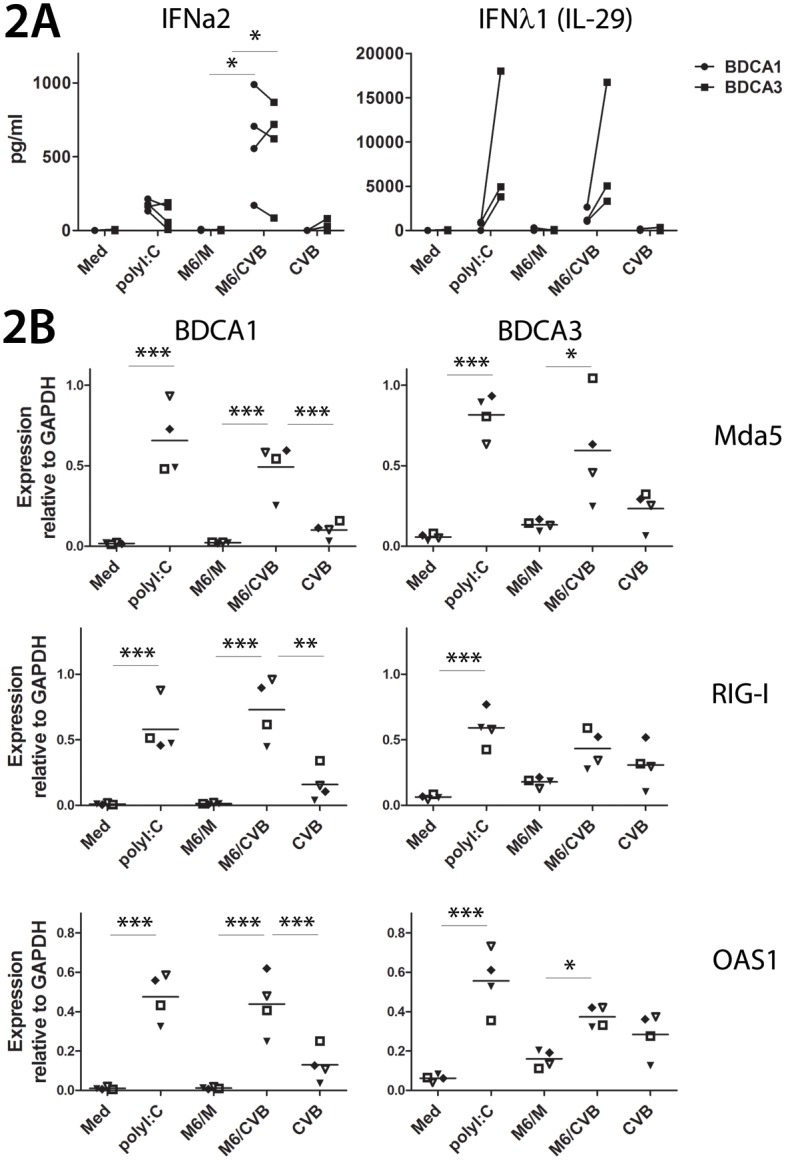
Type I and type III IFN responses are induced in mDC subsets upon encounter of CVB-infected cells. **A**) DCs were co-cultured with mock- or CVB-infected Min6 cells (M6/M or M6/CVB), infected with CVB3 (MOI 50), stimulated with poly I:C, or left unstimulated (medium; Med) and after o/n incubation supernatant was harvested and analyzed for production of IFN-α2 (left panel) or IFN-λ1 (right panel). Shown are data from 4 (IFN-α2) and 3 (IFN-λ1) different donors. **B**) DCs were stimulated as in A) and after 6 hours mRNA expression was analyzed by qPCR. Shown are data from 4 donors (B, corresponding symbols represent the same donor). * p<0.05, ** p<0.01, *** p<0.001 determined by ANOVA and post-hoc Tukey test.

To study the innate antiviral response of the mDC subsets we assessed the production of IFN-α2 and IFN-λ1. IFN-α2 was efficiently induced upon co-culture with CVB-infected, but not mock-infected β-cells in both mDC subsets, and reached similar levels (Fig. 2A, left panel). Strikingly, upon o/n culture the levels of IFN-α2 induced by virally-infected cells were approximately 3-fold higher on average compared to stimulation with poly I:C, a double-stranded RNA mimic which serves as a positive control. IFN-λ1 was also induced in both subsets upon stimulation with CVB-infected cells and poly I:C, yet BDCA3^+^ mDCs produced much more IFN-λ1 compared to BDCA1^+^ mDCs (3 to 6- fold higher induction, depending on the donor)(Fig. 2A, right panel). No major differences were observed for IFN-λ1 induction upon stimulation with poly I:C compared to CVB-infected Min6 cells.

Next, we used qPCR to study the induction of IFN-stimulated genes (ISGs) Mda5, RIG-I and OAS1 6 hours after stimulation. The use of human mRNA-specific primers enabled us to determine mRNA expression in human mDCs specifically, while not amplifying murine Min6 template. As shown in Fig. 2B, very low basal expression of ISGs Mda5, RIG-I and OAS1 was detected in unstimulated mDCs (medium, Med), although for all ISGs tested mRNA levels were higher in unstimulated BDCA3^+^ DCs than BDCA1^+^ mDCs. Stimulation with poly I:C for 6 hours resulted in major increase of the ISGs tested (Fig. 2B). Six hours co-culture with mock-infected cells (M6/M) had little effect on ISG-expression, whereas co-culture with CVB3-infected Min6 cells (M6/CVB) resulted in increased ISG expression, and reached comparable levels in BDCA1^+^ and BDCA3^+^ mDCs relative to the housekeeping gene GAPDH. For example Mda5 levels increased 11- to 33-fold over M6/M in BDCA1 mDCs and 2.5- to 7.3-fold in BDCA3 mDCs, depending on the donor (Fig. 2B). CVB3 is unable to productively infect BDCA1^+^ mDCs by direct infection [24], and only induced low ISG levels when high CVB3 titers (MOI 50) were used. In summary, these data show that type I and type III IFNs, as well as various ISGs, are efficiently induced in BDCA1^+^ and BDCA3^+^ mDCs upon encounter of CVB-infected, but not mock-infected β-cells.

### Uptake of CVB-infected β-cells results in phenotypic DC maturation and production of various cytokines and chemokines in BDCA1^+^ and BDCA3^+^ mDCs

**Fig 3:**
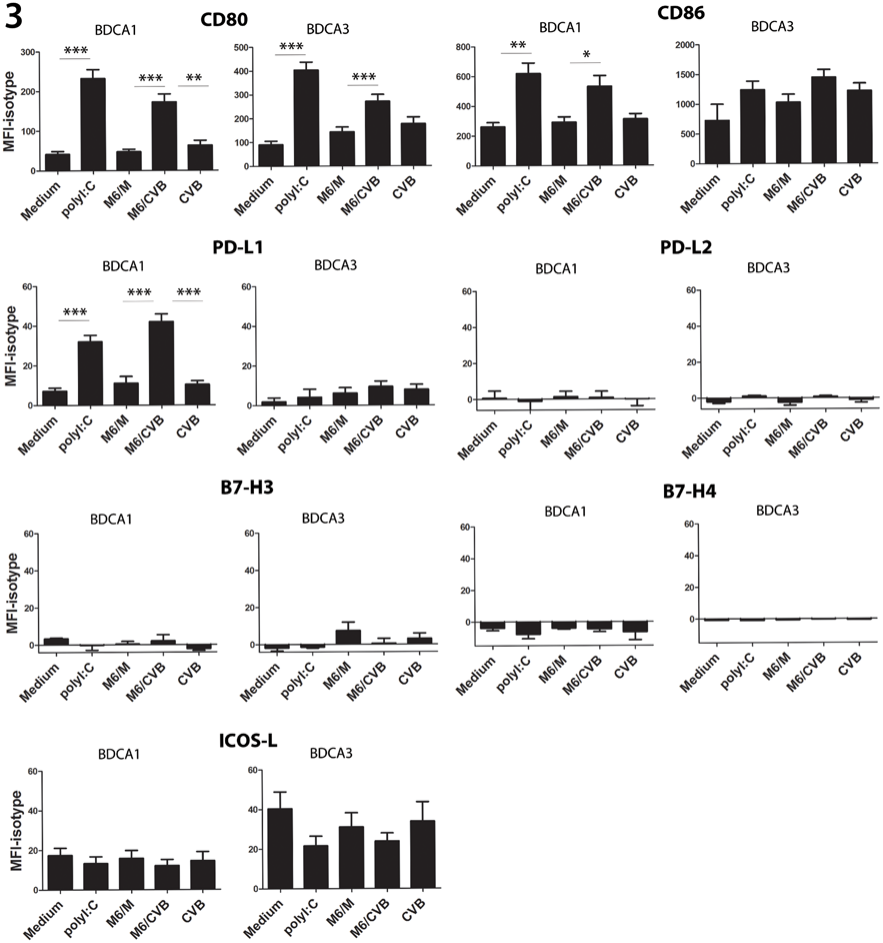
Phagocytosis of CVB-infected Min6 cells results in upregulation of co-stimulatory markers in BDCA1^+^ mDC and BDCA3^+^ mDC, and co-inhibitory marker PD-L1 in BDCA1^+^ mDCS. DCs were stimulated o/n as in Fig. 2A and subsequently analyzed for expression of indicated cell surface markers using flow cytometry. Shown are averages of mean fluorescence intensity (MFI)-isotype values of >3 donors + SEM. * p<0.05, ** p<0.01 determined by ANOVA and post-hoc Tukey test.

To investigate whether virus-infected cells induced mDC maturation we analyzed expression of co-stimulatory and co-inhibitory molecules from the B7 family [37] after o/n co-culture with Min6 cells. Co-culture with CVB3-infected Min6 cells resulted in significant increase of co-stimulatory markers CD80, CD86 in both mDC subsets (Fig. 3), as compared to mock-infected Min6 cells. The co-inhibitory marker PD-L1 was specifically induced upon encounter of CVB-infected cells in BDCA1^+^, but not BDCA3^+^ mDCs. Poly I:C, which was used as a positive control induced CD80, CD86 and PD-L1 to levels comparable to M6/CVB-stimulation. ICOS-L was expressed at low levels in both BDCA1^+^ and BDCA3^+^ mDCs, and expression was unaltered upon stimulation with mock- or CVB-infected Min6 cells. A modest decrease in ICOS-L expression was observed in poly I:C-stimulated BDCA3^+^ mDCs. PD-L2, B7-H3 and B7-H4 were not expressed, nor induced under any of the conditions tested (Fig. 3). Co-culture with mock-infected Min6 cells or stimulation with CVB3 (MOI 50) had no or only modest effects on expression of any of the cell surface markers tested in both mDC subsets (Fig. 3).

**Fig 4:**
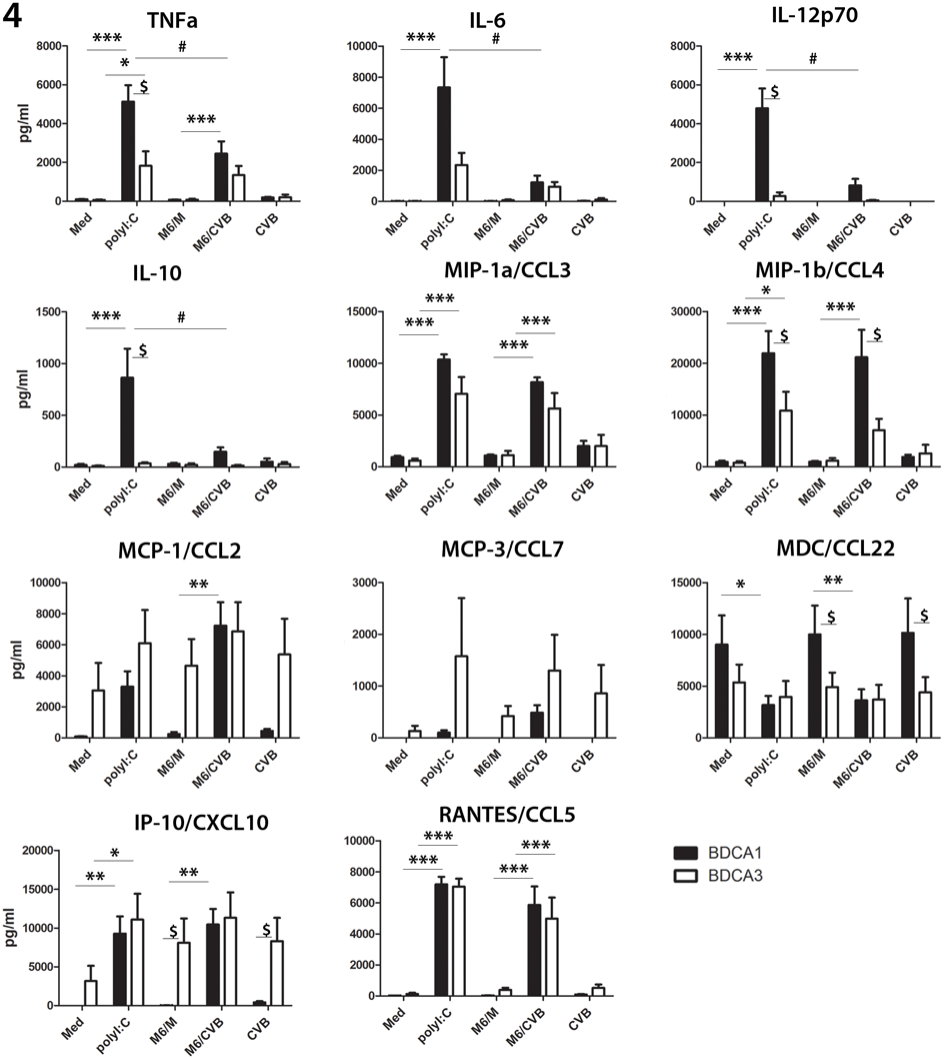
Cytokine and chemokine production in BDCA1^+^ and BDCA3^+^ mDCs upon encounter of mock- or virus-infected cells. DCs were stimulated o/n as in Fig. 2A and supernatant was analyzed for production of pro-inflammatory cytokines and chemokines. Data represent average of 4 independent experiments using different donors + SEM. * P<0.05, ** P<0.01, ***p<0.001 comparing M6/CVB versus M6/M or poly I:C versus medium; # p<0.05 Min6/CVB versus poly I:C; $ p<0.05 in BDCA1^+^ versus BDCA3^+^ mDCs with the same stimulus as determined by ANOVA and post-hoc Tukey test.

Next, we assessed production of a panel of pro-inflammatory cytokines and chemokines induced upon stimulation with poly I:C, mock- or CVB-infected Min6 cells, or CVB3 exposure. From mock or CVB3-infected Min6 cells alone no cytokines were detected in the human 22-plex, excluding that the observed cytokines are Min6-derived (S4 Fig). TNF-α and IL-6 were induced in both DC subsets upon poly I:C stimulation and co-culture with CVB-infected Min6 cells, but not mock-infected cells (Fig. 4), although higher levels of TNF-α were produced by BDCA1^+^ mDCs compared to BDCA3^+^ mDCs. IL-12p70 production by BDCA1^+^ mDCs was apparent in all donors upon stimulation with CVB-infected Min6 cells. Interestingly, little or no IL-12p70 was induced in BDCA3^+^ mDCs under the conditions tested. The anti-inflammatory cytokine IL-10 was induced in BDCA1^+^ mDCs upon stimulation with M6/CVB, yet like IL-12 was hardly produced by the BDCA3^+^ mDCs. Stimulation with poly I:C showed a similar trend for IL-12 and IL-10 with high levels found in BDCA1^+^ cultures but hardly any production in BDCA3^+^ cultures (Fig. 4).

Chemokines MIP-1α/CCL3 and MIP-1β/CCL4 were strongly induced in both mDC subsets upon M6/CVB-stimulation or poly I:C stimulation (Fig. 4), but were induced at much higher levels in BDCA1^+^ mDCs relative to BDCA3^+^ mDCs. For example, MIP-1β/CCL4 was induced 22-fold on average upon stimulation with M6/CVB in BDCA1^+^ mDCs, compared to a 6-fold increase in BDCA3^+^ mDCs. MCP-1/CCL2, MCP-3/CCL7 and IP-10/CXCL10 were highly induced in BDCA1^+^ mDCs upon poly I:C or M6/CVB stimulation compared to unstimulated DCs. Again the fold increase was much less for the BDCA3^+^ cultures, though expression under unstimulated conditions was higher for BDCA3^+^ DCs. RANTES/CCL5 was induced to similar extent in both subsets upon stimulation. The chemokine MDC was already produced by both subsets at unstimulated conditions and remained unaltered after stimulation of BDCA3^+^ mDCs whereas expression decreased in the BDCA1^+^ mDCs. In summary, these data indicate that both mDC subsets become activated upon encounter of CVB-infected Min6 cells, however the BDCA1^+^ mDC subset is a more potent producer of most pro-inflammatory and anti-inflammatory cytokines and chemokines tested.

### Both BDCA1^+^ and BDCA3^+^ mDCs efficiently induce Type 1 and suppress Type 2 responses upon encounter of CVB-infected β-cells

**Fig 5:**
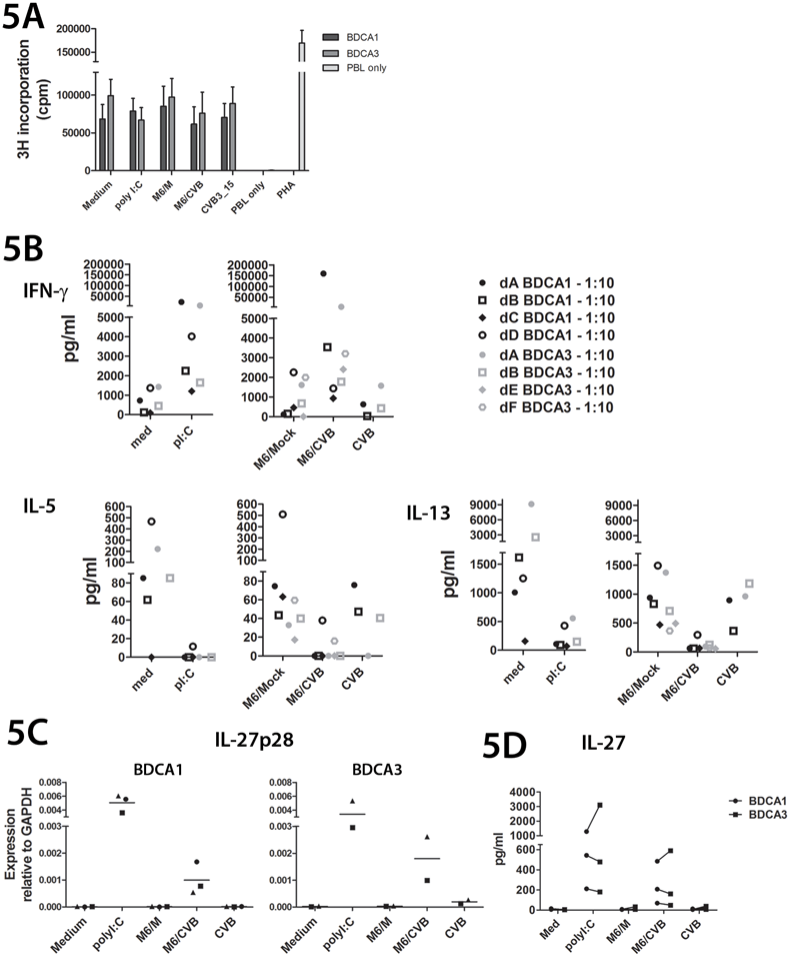
Induction of T cell proliferation and Th1 responses by BDCA1^+^ and BDCA3^+^ mDCs upon encounter of CVB-infected Min6 cells. **A**) DCs were stimulated o/n as in Fig. 2A, harvested and co-cultured with allogeneic PBLs in 1:10 DC:PBL ratio. PBLs without DCs (PBL only) or PBLs stimulated with phytohemagglutinin (PHA) were included as controls. Four days after start of co-culture proliferation was assessed using tritium incorporation. Cpm: counts per minute. **B**) Supernatant taken 48 hours after start of co-culture as in A) was analyzed for production of indicated cytokines. Each symbol represents one donor. * p<0.05, determined by ANOVA and post-hoc Tukey test. **C**) DCs were stimulated as in Fig. 2A, and 6 hours after stimulation mRNA expression was determined by qPCR. *** p<0.001 determined by ANOVA and post-hoc Tukey test. **D**) DCs were stimulated o/n as in Fig. 2A and IL27 production was assessed by ELISA. Each symbol represents one donor. Data are average of 2 independent experiments + SEM (A) or 2–4 donors from different experiments (B, C, D).

We compared the capacity of differentially stimulated mDC subsets to induce T cell proliferation and Type 1 or type 2 responses in allogeneic mixed lymphocyte reactions. Proliferation was assessed at day 4 and revealed that both DC subsets induce T cell proliferation in MLR in unstimulated conditions (medium), and that proliferation is not further enhanced upon stimulation with poly I:C, M6/M or M6/CVB (Fig. 5A). T cell cytokine analysis revealed that poly I:C and M6/CVB stimulated DCs induced production of Type 1 cytokine IFN-γ while simultaneously strongly suppressing Type 2 cytokines IL-5 and IL-13 (Fig. 5B). This was corroborated by qPCR analysis of IFN-γ and IL-5 in BDCA1^+^ and BDCA3^+^ MLR samples (S5 Fig). There were no significant differences in IFN-γ induction capacity between both mDC subsets. No consistent induction of Th17 cells was observed for BDCA1^+^ nor BDCA3^+^ mDCs in allogeneic MLR under the conditions tested (S6 Fig). These data indicate that BDCA1^+^ and BDCA3^+^ mDCs are equally efficient in inducing Type 1 responses upon encounter of enterovirus-infected cells, despite lower uptake capacity of BDCA3^+^ mDCs and lower production of IL-12p70.

Besides IL-12, other cytokines are known that may (synergistically) induce Type 1 responses, such as the IL-12-like cytokine IL-27 [38–40]. We hypothesized that IL-27 may be produced in larger quantities by BDCA3^+^ mDCs than BDCA1^+^ mDCs, thereby facilitating efficient Type 1 induction in BDCA3^+^ MLR cultures, despite much lower IL-12 production. Both BDCA1^+^ and BDCA3^+^ mDCs strongly expressed IL-27p28 (IL27A) mRNA upon stimulation with M6/CVB or poly I:C, but not M6/M (Fig. 5C). EBI3 (IL27B) was expressed at higher levels compared to IL27p28 in unstimulated cultures and expression was unaltered upon stimulation with M6/M or M6/CVB (S7 Fig). IL-27 ELISA corroborated our findings on IL-27p28 mRNA induction in BDCA1^+^ and BDCA3^+^ mDCs (Fig. 5D). Since IL-27 is equally induced by BDCA1^+^ and BDCA3^+^ mDCs, it is not likely the factor driving Type 1 polarization in BDCA3^+^ cultures as a substitute of IL-12 in BDCA1^+^cultures.

## Discussion

The precise pathophysiology resulting in T cell activation during autoimmunity development remains unclear; however, viral infections can trigger the development of autoimmunity [2, 7]. Distinct subsets of mDCs could differentially influence T cell activation upon encounter of virus-infected cells. However, although mRNA profiling studies predict functional differences between human BDCA1^+^ and BDCA3^+^ mDC, relatively few studies provide experimental evidence for this in response to virus-infected cells. Here, we report on the response of human BDCA1^+^ and BDCA3^+^ mDC to mock- versus CVB-infected β-cells, conditions mimicking the physiological encounter of virus-infected cells and APCs. Our data show that BDCA1^+^ mDC more efficiently engulf mock- or CVB-infected Min6 material compared to BDCA3^+^ mDCs. Uptake of virus-infected β-cells resulted in induction of co-stimulatory molecules, production of type I and III IFNs and ISG-induction. Similar type I IFN secretion was observed in both mDC subsets, yet BDCA3^+^ mDCs are superior in IFN-λ production. BDCA1^+^ mDCs were more potent inducers of co-inhibitory PD-L1 and produced most pro-inflammatory cytokines and chemokines, with a striking difference in IL-12p70 and IL-10, which are hardly produced by BDCA3^+^ mDCs. Our data indicate that the mDC subsets exhibit distinct response patterns upon encounter of enterovirus-infected cells, but both promoted Th1 T cell responses in an allogeneic MLR.

We observed efficient uptake of mock- as well as CVB-infected Min6 cells. The reason for efficient uptake of mock-infected Min6 cells is unknown, but could relate to enhanced molecular signals that mediate phagocytosis as previously discussed [34]. The differences in uptake capacity of Min6 material by human DC subsets is in accordance with data from Tel *et al*. who showed that BDCA1^+^ mDCs far more efficiently engulf particulate antigens compared to other human DC subsets [41]. Of note, no direct linear correlation was observed between amount of virus-infected β-cell uptake and subsequent response; e.g. despite a lower uptake BDCA3^+^ DCs produce more type III IFNs and show equal type I IFN responses. Different uptake mechanisms of cellular debris, different expression levels of PRRs and/or downstream signaling molecules, or a combination of both may cause strong BDCA3^+^ DC activation, despite lower uptake. For example, higher expression levels of the double-stranded RNA sensor TLR3 in BDCA3^+^ mDCs [28, 29] may cause differential downstream responses as well as different synergistic responses in BDCA1^+^ and BDCA3^+^ mDC. Additionally, BDCA3^+^ mDCs may more specifically engulf “pathogen-associated material”, through expression of specialized uptake receptors like the C-type lectin CLEC9A [27, 42]. Importantly, regardless the differences that may exist between BDCA1^+^ and BDCA3^+^ DC subsets in amount of uptake and cargo selection, our data clearly show that both subsets induce distinct, but partially overlapping antiviral responses.

In our current studies we used murine insulinoma cells to investigate the response of human primary mDC subsets to CVB-infected β-cells. In our previous studies using moDC we did not observe clear differences in the responses to infected murine Min6 cells, porcine islets of Langerhans or human islets of Langerhans [34]. Nevertheless, analysis of the response of fresh human DCs to infected human β-cells or primary human islets of Langerhans should confirm our findings in future studies. The current experiments show clear DC-activation following uptake of Min6 cells. It remains to be determined to which extent the viral components and the dead or dying cells themselves contribute to the observed responses. It is known that virus-infected cells or apoptotic cells containing synthetic double-stranded RNA (dsRNA) lead to strong DC activation, whereas apoptotic cells without dsRNA do not [43]. However, because CVB-induced cell death results in release of endogenous ‘danger signals’ such as HMGB1 [44], virus-induced cell death may contribute to the observed effects. Future experiments in which cells are exposed to dead or dying cells not caused by virus infection should help to further elucidate this issue.

BDCA1^+^ mDCs are considered the human counterparts of murine CD8α^-^ DCs, which according to previous studies are poor inducers of type I IFNs, and IL-12p70 and relatively poor Th1 inducers [45]. This may, however, differ between mice and human, and could depend on the particular stimulation. Our study reveals that upon encounter of enterovirus-infected β-cells, BDCA1^+^ mDCs produce similar amounts of the type I IFN-α2 compared to BDCA3^+^ mDCs that are considered the human counterpart of murine CD8α^+^ DC. Furthermore, also the extent of ISG induction is comparable between BDCA1^+^ and BDCA3^+^ mDCs, indicating that both subsets can play a critical role in local anti-viral responses *in vivo*. Lauterbach and colleagues [46] showed that especially BDCA3^+^ mDCs stimulated with the TLR-ligand poly I:C produce IFN-λ1. Our data confirm these findings and show that BDCA3^+^ mDCs were also far more efficient inducers of IFN-λ1 upon encounter with CVB-infected β-cells. Interestingly, a recent study showed that islets of Langerhans are protected from enterovirus infection after treatment with IFN-λ [47]. Whether and how IFN-λ influences autoimmune responses against β-cells remains to be determined.

Our study revealed that upon encounter of virus-infected, but not mock-infected cells, BDCA1^+^ mDCs showed higher induction of pro-inflammatory cytokines and chemokines MIP-1α/CCL3, MIP-1β/CCL4 and MCP1/CCL2 compared to BDCA3^+^ mDCs, which is in accordance with findings from others [28]. One major difference between our results and those of Hémont *et al*., is the absence of MCP-3/CCL7 in TLR-stimulated BDCA1^+^ and BDCA3^+^ mDC cultures in their studies, whereas for BDCA1^+^ mDCs we find that MCP-3 is induced to modest extent upon poly I:C-stimulation, and is much stronger induced upon encounter of CVB-infected cells. This shows that differential responses are induced upon encounter of purified TLR-ligands compared to the more physiological setting of encountering virus-infected cells. Interestingly, MCP-3 expression is elevated in monocytes from type 1 diabetes patients compared to healthy donors or type 2 diabetes patients [48]; and MCP-3 is also elevated in serum of autoantibody-positive individuals [49]; indicating a possibly pathological role for this cytokine in autoimmune diabetes pathogenesis. The observation that BDCA1^+^ DCs are more potent chemokines producers, whereas BDCA3^+^ DCs are superior in type III IFN production suggests that besides overlapping functions, these mDC subsets do have more specialized functions in e.g. chemoattraction of other immune cells versus induction of antiviral responses through IFN-λ. Further in depth analysis is required to fully elucidate the complementary and differential functions of human BDCA1^+^ mDCs, BDCA3^+^ mDCs and their cross-talk in (auto)immunity.

Furthermore, IL-12p70 production was low/absent in BDCA3^+^ mDCs stimulated with either poly I:C or CVB-infected cells. Similar observations were done by Jongbloed *et al*. and Hémont *et al*. [28, 29]. Despite the much lower induction of IL12-p70 by BDCA3^+^ mDCs, they induce Th1 responses (IFN-γ) and suppress Th2 responses (IL-5 and IL-13) equally well compared to BDCA1^+^ mDCs under the conditions tested. Previous studies reported alternative IL-12-independent mechanisms to achieve Th1 polarization [50–52], for example via the induction of type I IFNs [51]. Yet we did not find differences in IFN-α production between both mDC subsets. Furthermore, no major differences between BDCA1^+^ and BDCA3^+^ mDCs were observed in induction of IL-27, an IL-12 like cytokine with Th1 inducing capacities [38–40]. An alternative mechanism that could facilitate efficient Th1 induction in BDCA3^+^ MLR cultures could be the ratio between co-stimulatory and co-inhibitory markers. The absence of PD-L1 on BDCA3^+^ may steer towards Th1 phenotype in that case. Alternatively, the much lower production of IL-10 by BDCA3^+^ mDCs may support Th1 skewing. Additionally, DCs matured in the presence of IFN-λ induced lower IL-13 production and increased IFN-γ secretion [53, 54]. Thus, the observed IFN-λ production in BDCA3^+^ mDCs could contribute to decreased Th2 cytokines and increased IFN-γ induction in our experiments. Furthermore, Poulin *et al*. showed that BDCA3^+^ mDCs only produce IL-12 after CD40 ligation [55]. Thus, BDCA3^+^ mDCs in MLR cultures may produce IL-12 after CD40 ligation by T cells, resulting in enhanced Th1 induction. In summary, BDCA3^+^ mDCs are as efficient as BDCA1^+^ mDCs to induce Th1 polarization, but employ a different mechanism that requires further in depth analysis to elucidate.

The data presented yield valuable new insight into the physiological responses of freshly isolated primary human blood DCs. The mDC subsets studied here can also be detected in different human lymph nodes [56]. Despite the complexity of the experimental set up and the use of human donors, similar trends are observed in essentially all donors. The data do, however, not always reach statistical significance, mainly because the difference in the strength of the responses in the inherently variable human population. Nevertheless, the data clearly show that DCs exposed to virus-infected, but not mock-infected β-cells give rise to potent Th1 responses. These findings support the hypothesis that the co-presence of self-antigens in the virus infected cells could result in initiation and/or acceleration of autoreactive responses by such activated DCs in susceptible individuals [7, 8]. Additionally, whether and which differences exist between DC subsets from healthy donors and type 1 diabetes patients in the response enterovirus-infected cells remains to be determined.

## Supporting Information

S1 Fig Gating strategy to analyze uptake and DC phenotype specifically in viable single cells. (TIF)

S2 Fig Appearance of Min6 cells 48 hours after infection. Magnification 40x.
(TIF)

S3 Fig Confocal analysis of BDCA1^+^ DCs co-cultured with Min6 cells. DCs were co-cultured with PKH67-labeled (green) mock- or CVB-infected Min6 cells for 18hrs and were subsequently harvested, adhered onto poly-L-Lysine-coated coverslips, stained for MHC class II (red) and DAPI (nuclear stain, blue) and analyzed using confocal laser scanning microscopy. White arrows indicate examples of DCs that have phagocytosed Min6 material.
(TIF)

S4 Fig No detection of human cytokines from Min6 cultures. Min6 cells were mock- or CVB-infected and after 18 hrs supernatant was harvested and analysed for indicated cytokines. Supernatant from BDCA1^+^ DCs stimulated with poly I:C is shown as a positive control.
(TIF)

S5 Fig mRNA induction of IFNgamma and reduction of IL-5 in MLR cultures from stimulated BDCA1^+^ and BDCA3^+^ DCs. MLRs were performed as in Fig. 5A. After 48 hrs total RNA was harvested as described and analyzed for expression of IFNgamma and IL-5.
(TIF)

S6 Fig Production of IL-17 in MLR cultures of BDCA1 and BDCA3 DCs. Supernatant taken 48 hours after start of co-culture as in Fig. 5A) was analyzed for production of IL-17A.
(TIF)

S7 Fig No induction of EBI3 upon co-culture of DC subsets with mock- or CVB-infected Min6 cells. DCs were stimulated as in Fig. 2A, and 6 hours after stimulation mRNA expression was determined by qPCR. *** p<0.001 determined by ANOVA and post-hoc Tukey test.
(TIF)
