# Supplementary figures and images for: Enterovirus-Infected β-Cells Induce Distinct Response Patterns in BDCA1+ and BDCA3+ Human Dendritic Cells
Source: PLoS One. 2015 Mar 25;10(3):e0121670. doi: 10.1371/journal.pone.0121670 (PMC4373773; doi:10.1371/journal.pone.0121670)

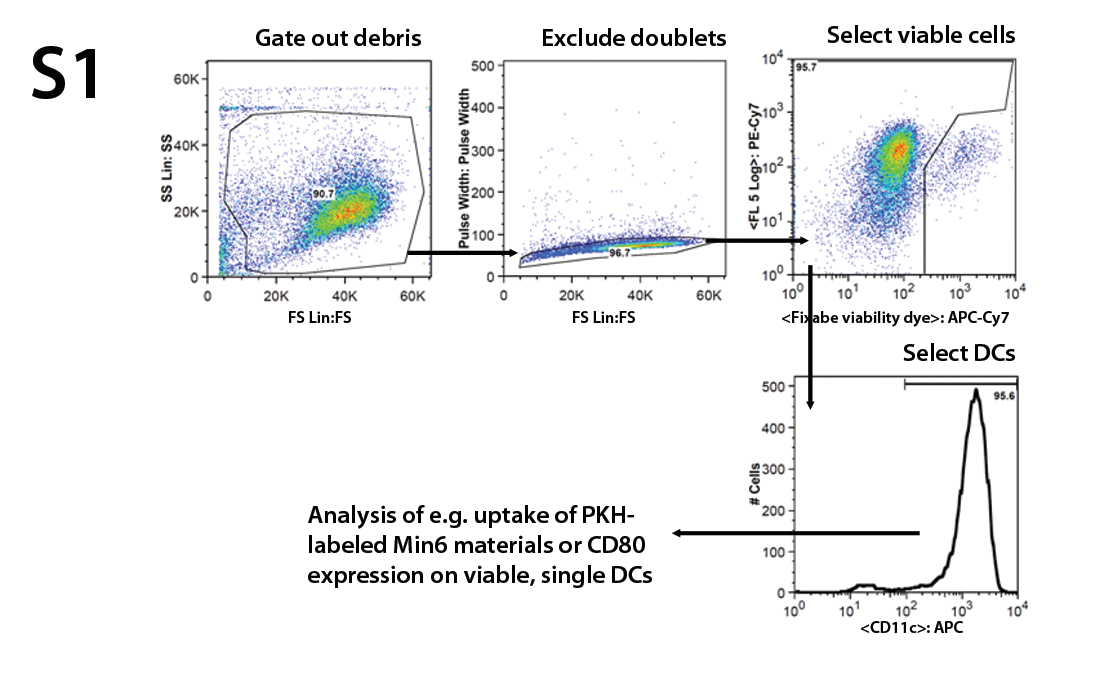

Supplement: S1 Fig — (TIF) [file pone.0121670.s001.tif]

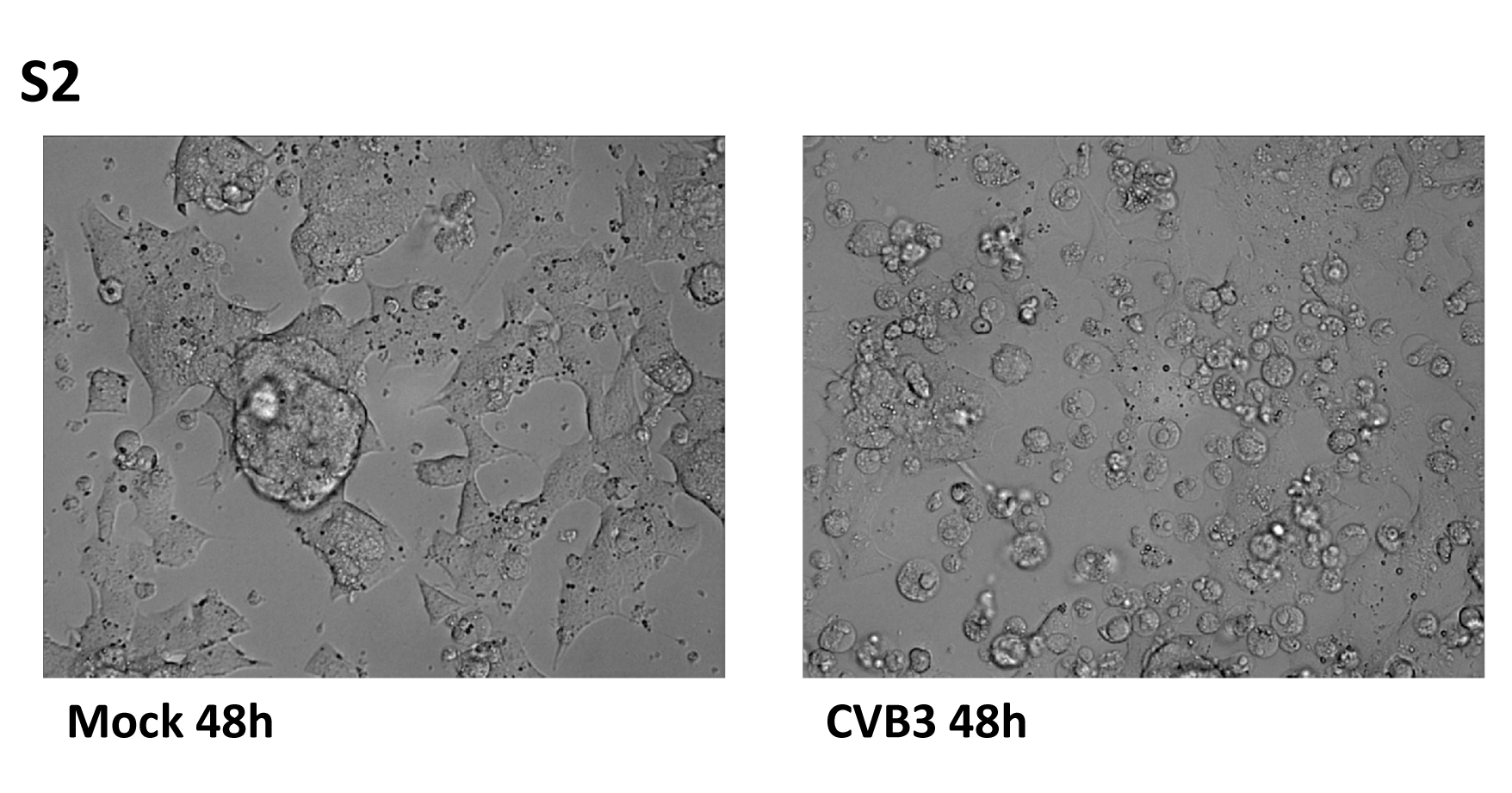

Supplement: S2 Fig — Magnification 40x. (TIF) [file pone.0121670.s002.tif]

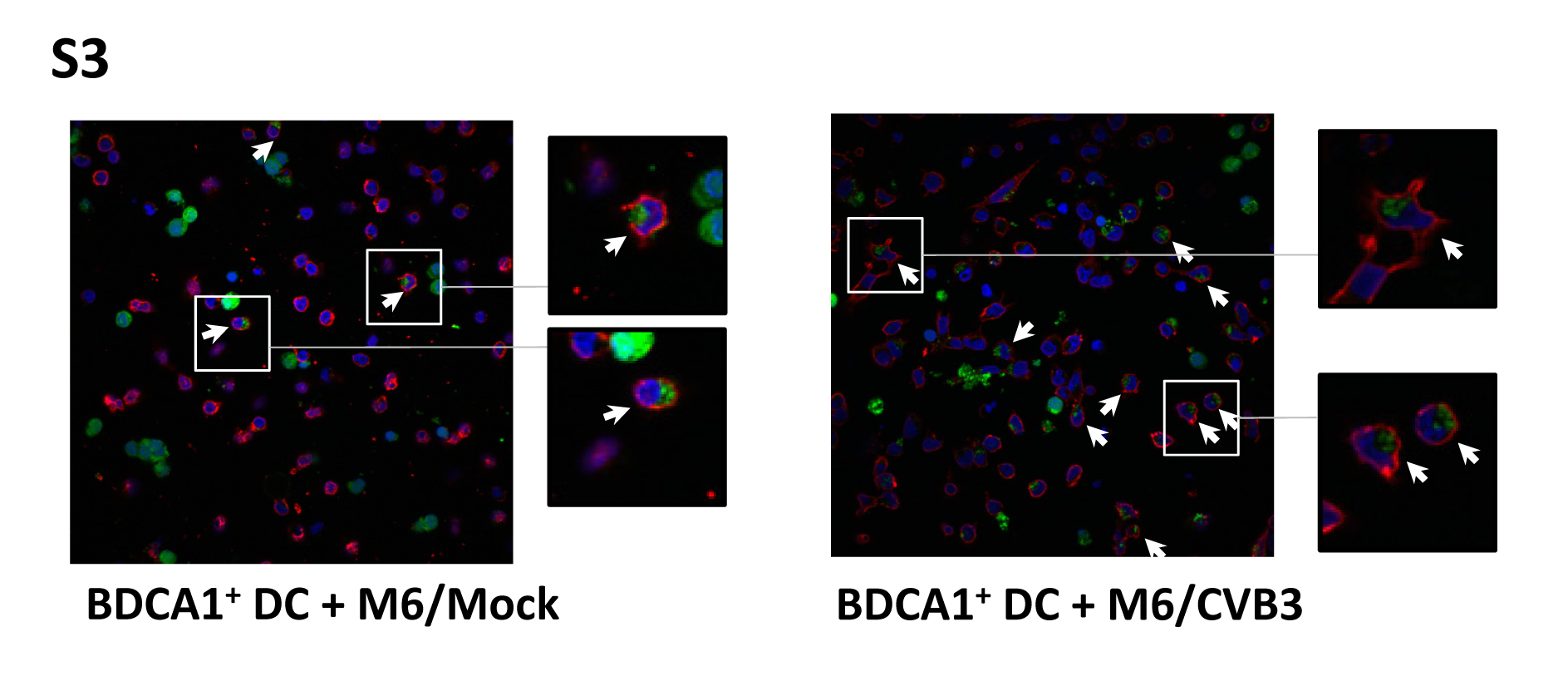

Supplement: S3 Fig — DCs were co-cultured with PKH67-labeled (green) mock- or CVB-infected Min6 cells for 18hrs and were subsequently harvested, adhered onto poly-L-Lysine-coated coverslips, stained for MHC class II (red) and DAPI (nuclear stain, blue) and analyzed using confocal laser scanning microscopy. White arrows indicate examples of DCs that have phagocytosed Min6 material. (TIF) [file pone.0121670.s003.tif]

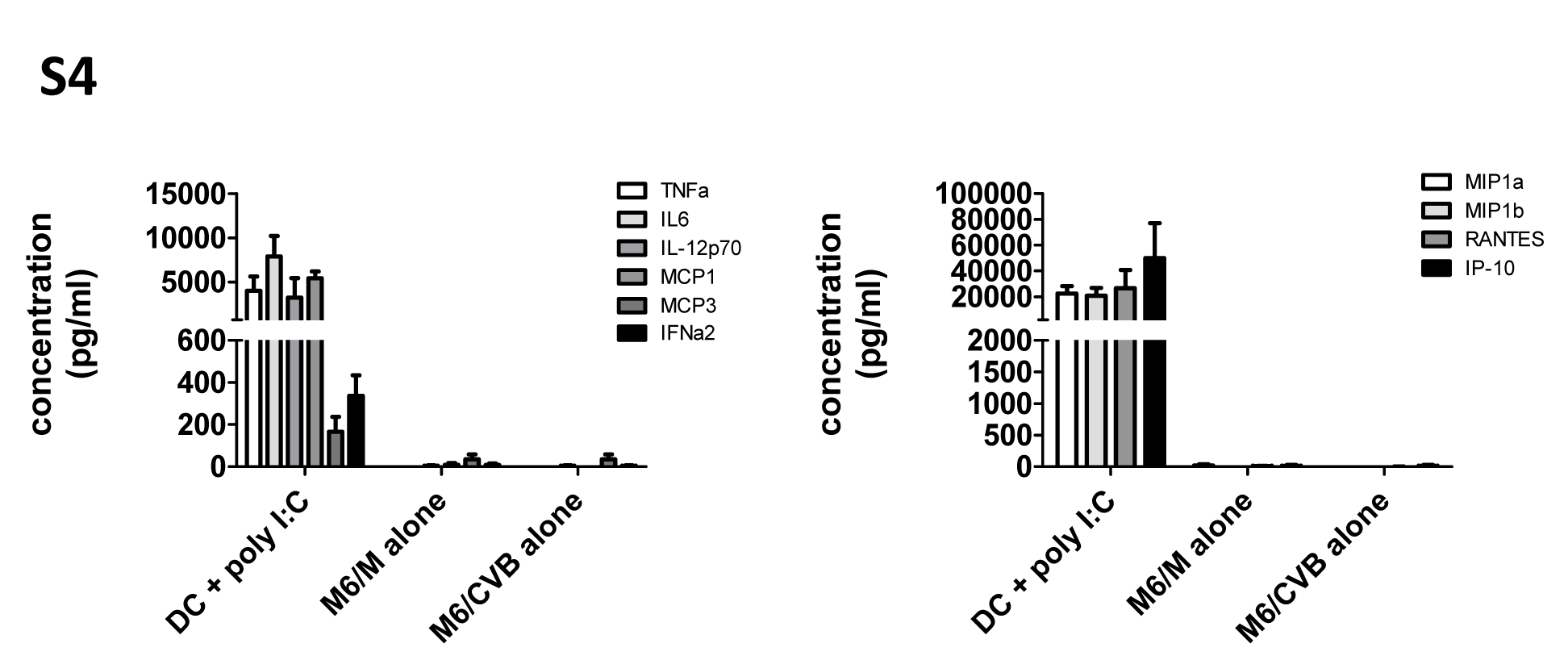

Supplement: S4 Fig — Min6 cells were mock- or CVB-infected and after 18 hrs supernatant was harvested and analysed for indicated cytokines. Supernatant from BDCA1+ DCs stimulated with poly I:C is shown as a positive control. (TIF) [file pone.0121670.s004.tif]

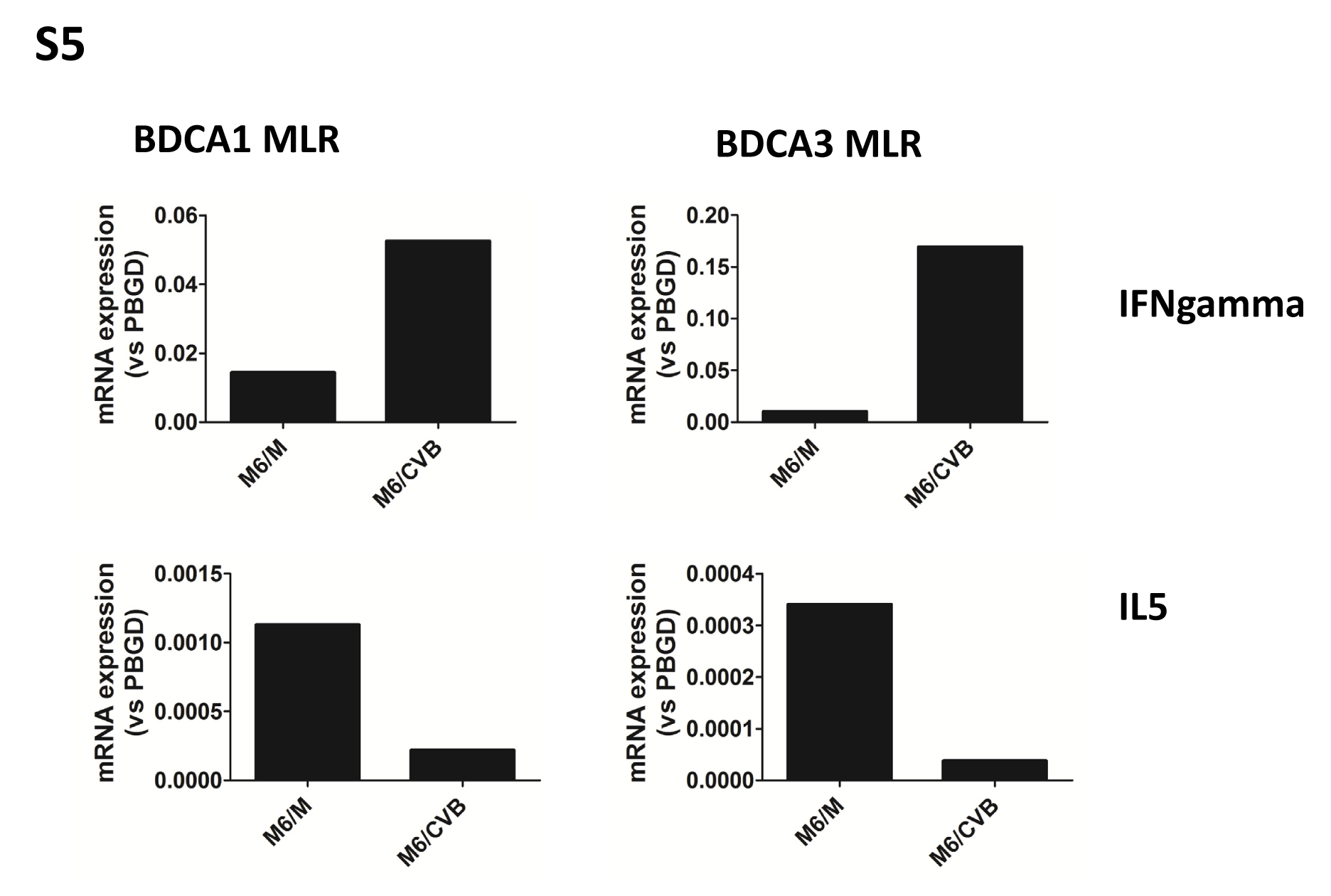

Supplement: S5 Fig — MLRs were performed as in Fig. 5A. After 48 hrs total RNA was harvested as described and analyzed for expression of IFNgamma and IL-5. (TIF) [file pone.0121670.s005.tif]

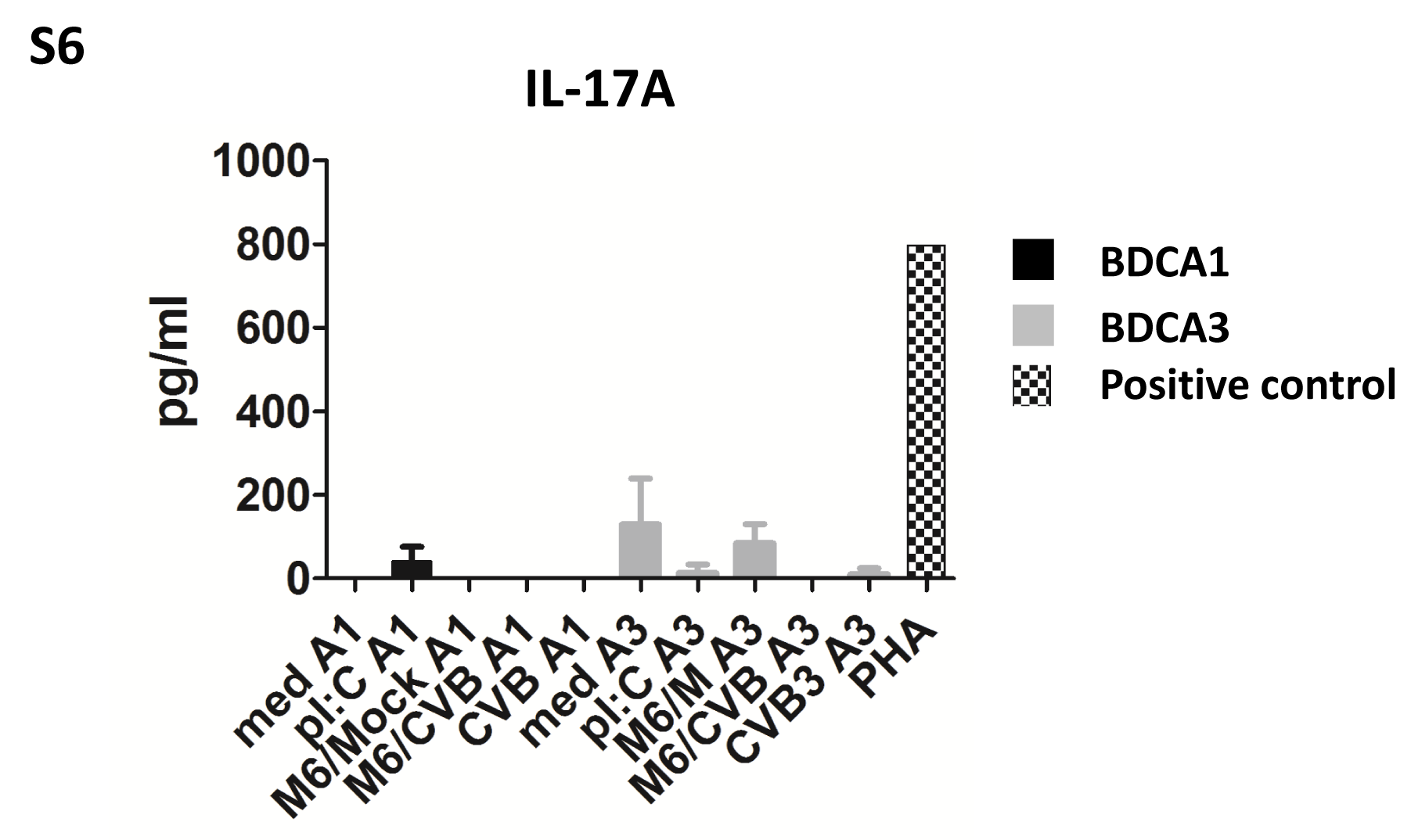

Supplement: S6 Fig — Supernatant taken 48 hours after start of co-culture as in Fig. 5A) was analyzed for production of IL-17A. (TIF) [file pone.0121670.s006.tif]

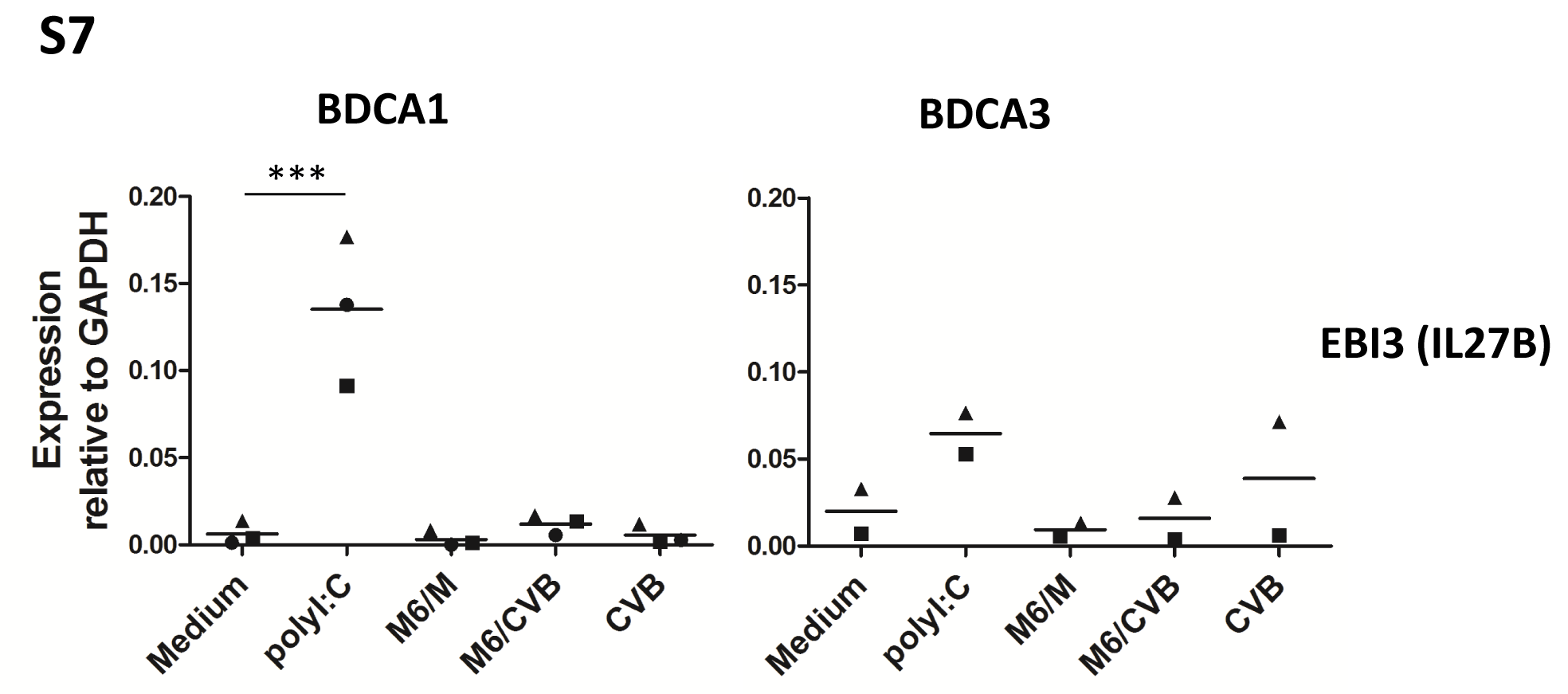

Supplement: S7 Fig — DCs were stimulated as in Fig. 2A, and 6 hours after stimulation mRNA expression was determined by qPCR. *** p<0.001 determined by ANOVA and post-hoc Tukey test. (TIF) [file pone.0121670.s007.tif]
